# Extracts and Gleanings

**Published:** 1872-06

**Authors:** 


### PART II.

### Abridged Extracts and Gleanings from our Exchanges

*MULTUM IN PARVO.*

---

For the benefit of the readers of The Companion, we transcribe the subjoined recipes from a recent work ("The Medical Adviser") of the late Dr. Thompson, of Tennessee. These we have used almost daily for twelve months, and have found them of sterling merit to the ousy practitioner :

Fever Syrup.—

| | |
|---|---|
| R.—Syrup Rhubarb, . . . . | oz. iv, |
| Tinct. Valerian, . . . . . | oz. ij. |
| Oil Sassafras, . . . . . | gtts. xx. |
| Piperin, . . . . . . | grs. x. |
| Sup. Carb. Soda, . . . . | grs. xx. |

First mix the oil and piperin thoroughly with the syrup before adding the valerian.

Dose, from a teaspoonful to a tablespoonful. Best given in double the amount of sweet milk. The author says "this compound will answer in more cases of sickness than any other I have ever known."

Highly recommended in fevers of all grades, and particularly in typhoid affections. The therapeutical properties of each ingredient will suggest to the mind of every intelligent physician the indications that may be made in different diseases.

Chloroform Liniment.—

| | |
|---|---|
| R.—Spts. Nitre, . . . . . . | oz. iv. |
| Spts. Amonia and Chloroform aa. . . | oz. i. |
| Camphor, Oil Sassafras, Oil Juniper, aa. | oz. ss.—M. |

We have found this a good liniment for general use, and well adapted to allay pain or spinal irritation in all febrile ailments.

ALTERATIVE SYRUP.—

| | |
|---|---|
| R.—Fluid Ext. Sarsap., . . . . . | oz. vi. |
| Tinct. Valerian, Syr. Rhubarb, aa. . | oz. ij. |
| Piperin, . . . . . . . | grs. xxx. |
| Iod. Potash, . . . . . . | dr. vi.—M. |

Dose, from a teaspoonful to a tablespoonful after meals, to be taken in milk. The above will rarely fail to accomplish all that may be expected of an alterative.

ANTI-SYPHILITITIC SYRUP.—

| | |
|---|---|
| R.—Fever Syrup, Syr. Sarsap. Comp., aa. | oz. viij. |
| Iodide Potas., . . . . . | oz. i. |
| Corrosive Sublimate, . . . . | grs. xxx.—M. |

We indorse our author in his high recommendations of the above in syphilis. Dose a tablespoonful after meals.

FOR LEUCORRHŒA.—

| | |
|---|---|
| R.—Bal. Copaiv., . . . . . . | oz. ss. |
| Spts. Nit., . . . . . . | oz. ij. |
| Spts. Lav. Comp., . . . . . | oz. ss. |
| Simp. Syrup, . . . . . . | oz. iij.—M. |

S.—Dose, teaspoonful from three to five times daily. We have found this a good plan of giving the balsam, and withal very efficient in leucorrhœa. J. KNOX HODGE, M. D.

Holly Springs, Ark.

CHRONIC DIARRHŒA, SEQUEL OF TYPHOID FEVER.—In a case of this character, Professor N. S. Davis (Chicago Medical Examiner, Feb. 1, '72) says:

There was much emaciation; haggard expression; quick, weak pulse; much abdominal tympanites and distention; and still from eight to twelve intestinal evacuations of thin, reddish-brown stools, often mixed with blood. He was then put on the use of syrup of ipecac and tincture of opium, equal parts mixed, half a fluid drachm every two hours. The dose was subsequently increased to nearly a drachm. During the first four or five days this produced a decidedly favorable influence, lessening the number of discharges, and improving their quality. After that time it began to lose its benefical effect, and it was continued at intervals of every four hours, and a pill of nitrate of silver, one-third of a grain with pulverized opium, two grains, was given between—making them two hours apart. From that time he began to improve, and after about two weeks his discharges were reduced to one in the twenty-four hours, but it was still thin. The interval between the doses of medicine was lengthened to six hours, at which rate he has continued to the present time, December 30, 1871.

He is now able to sit up part of the day; is gaining in flesh; appetite good; but feet become œdematous when dependent. The Professor explained that the patient, though steadily improving, could not be left safely without further treatment. The ulcerated patches in the mucous membrane were not entirely cicatrized, and if treatment is discontinued too soon the diarrhœa will increase again. The patient was directed to adhere to a bland nutritious diet; take one of the pills of opium and nitrate of silver before breakfast, dinner and supper, and two-thirds of a teaspoonful of the mixture of syrup of ipecac and tincture of opium at bed time.

NOTE.—January 30, 1872.—At this date the patient is apparently well; bowels regular and passages natural, suffering only from general debility. He has taken no medicine for the last week.

STYPTIC COTTON.—Dr. J. Cummisky (Philadelphia Medical Times, January, '72) gives the following formula of Dr. Ehrle:

Take cotton of the best quality; boil it in a weak solution of soda (four per cent.) for about an hour; wash with cold water; press out and dry. Then steep the cotton in a solution of the chloride of iron (diluted one-third); press and air dry; after which pick to pieces. And then adds: This prepared cotton is of a yellowish brown color, and requires to be kept protected from the air, as it absorbs moisture very rapidly, owing to the delinquescent character of the iron salt which it contains. Having prepared some for experiment a few months ago, after using it occurred to me that if the sol. ferri subsulph. were used in its preparation, instead of the sol. ferri chlor., a superior article would be made, inasmuch as the cotton would not then absorb moisture, and would not need the care to protect it which this did. I made some accordingly, and found it quite as efficient a styptic, and entirely free from the objection referred to. It is of a light straw color, and perfectly dry to the touch. It is easily carried, and ever ready for use. It acts chemically and mechanically—chemically through the iron salt which it contains, and mechanically by means of its bulk and closely-pressed fibres. It has been in constant use at the St. Mary's Hospital for some months, and is looked upon by the surgeons there as a valuable addition to their armamentarium.

Chronic Catarrh of the Uterus.—The following case is reported by Dr. G. Kaiser in the Cincinnati Medical News:

In the latter part of December, 1870, I was called to attend upon Mrs. J. D. S., æt. twenty-nine years, married, and the mother of two children, the youngest about five years old. Mrs. S. stated that she had been in bad health since the birth of her second child, which she attributed to the last of her labor pains with this child, which were unusually severe and protracted; that she was now in her fifth month of pregnancy, and complained of pain in her head, back, and hips, with shooting and throbbing pains throughout the pelvis; had discharges from the vagina which stained her clothes, and was at times slightly mixed with blood; had very frequent nervous hysteric spells, impaired digestion, and had been confined to her bed for nearly twelve months. She also complained of pains resembling labor pains; and that she had had medical attendance for nearly three years without deriving any relief. With this view I made a vaginal examination, and detected the following, viz: ulceration of the os uteri, with muco-purulent discharges, hyperæsthesis of the portio-vaginalis, which was swollen, hypertrophied, and of a dark red color. The introduction of the speculum caused her great pain, and brought on her a nervous hysteric spell. Her nutritive functions were deranged. Her urine contained a deposit of lithates. Her bowels were in general regular, but her general condition extremely nervous and debilitated.

Having before me a great variety of symptoms, I directed my attention and treatment mainly to the uterus, which I placed as the seat of the disease, taking in consideration her nervous system.

The womb is supplied with nerves by the two great divisions of the nervous system, viz: the cerebro-spinal axis, and the trisplanchic nerves. The first preside over animal life, whilst the second are essential to organic existence. The pain in the back and head, the result of uterine disease, is conveyed through the cerebro-spinal axis, while the organic derangements, such as are observed to occur in the stomach, heart, and digestive organs generally, are due to the action of the ganglionic department. The feature of lithate deposit in the urine is one of the evidences of impaired digestion.

Under the circumstances, the following was my treatment: Ordered the vagina to be well clansed morning and evening, with an infusion of chamomile flowers, milk-warm, by means of a Davidson syringe, and to be continued until further orders. Also

R.—Potass. Bromid., . . . . . . oz. ss.
Chloral Hydratis, . . . . . dr. j.
Syr. et aqua menth. pip, aa. . . . oz. iij.
S.—One desertspoonful morning and evening.

R.—Quin. Sulph., . . . . . . dr. ss.
Massæ. Pillul., . . . . . . dr. ss.
Ferri Carbon., . . . . . . dr. ss.
Ext. Gentian, . . . . . . dr. j.—M.
Ft. pill No. xxx.
S.—One pill three times a day.

January 12.—Discharge per vagina only slightly reduced; exploration with the finger still produces great pain in the portio-vaginalis; general condition slightly better. Ordered the vagina to be syringed with infusion of belladonna, half a pint with one teaspoonful aquæ laurocerasor, morning and evening; the last pills to be repeated, and the following powders:

R.—Pulv. Castorei, . . . . . . dr. j.
Pulv. Rad. Valerian, . . . . . dr. j.
Sacch. Lactis, . . . . . . dr. ij.—M.
Mt. Pulves lx.
S.—One powder three times a day.

January 28.—Found patient much improved; the tenderness in the vagina has disappeared.

February 6.—Made a painless vaginal examination with the speculum. The os had nearly a normal appearance, with the exception of a small ulcerated spot about a quarter of an inch in diameter, at the anterior median line of the os, to which I applied slightly and gently actual cautery. Ordered the last injection and medicine to be continued.

March 12.—Discharged patient well. The ulcers above described were entirely healed; no discharge of any consequence from the vagina. Patient has gained much in strength, and is walking over the house.

April 26, 10 o'clock, A. M.—I delivered her of a large, healthy female child, of 11¼ pounds in weight. There was adhesion of the placenta to the fundus uteri, which I removed with little trouble. The day following the delivery she was taken with fever, which disappeared under little modification, and I have the satisfaction of saying that she is now fully recovered, and able to attend to her household duties herself without the least inconvenience whatever.

In the whole course of my medical study and practice I do not know of any case that has given me more personal satisfaction than the undoubted radical cure of this patient. The points here given are only a synopsis of the general complaints; it would take many pages to enumerate them all. I attribute my main success in restoring this patient to health to her strict observance of my directions, and especially to the thorough cleansing of the the affected parts by means of the syringe, thereby stimulating the parts to a healthy and restorative action. In conclusion, I will state that Mrs. S. had never before used, neither had she ever been advised to use, a vaginal syringe.—*Medical and Surgical Reporter*, *Feb.*, '72.

Arsenic in Dyspepsia.—Dr. Lawbaugh (Medical and Surgical Reporter, Feb. 3, '72) gives the following case:

R. S., æt. 31, applied to me for relief from attacks of severe pain accompanied with vomiting, generally occurring on hour or so after meals, but frequently several times during the night; he could obtain but little sleep.

His countenance was natural, but his mind dejected; the tongue clean; bowels slightly constipated; urine normal. Nothing could be detected on palpation of the abdomen, but slight pain was produced in the epigastric region. Various forms of medication had been tried, but without much relief.

An easily digested diet was ordered, with two drops of Fowler's solution a short time before each meal. Treatment continued for three weeks with perfect relief from all painful symptoms.

Arsenic in Irritable Stomach.—Dr. A. J. Lawbaugh (Medical and Surgical Reporter, Feb. 3, '72) reporting a case of irritable stomach, says:

The Symptoms noted were: A general feeling of hunger, accompanied by a sinking sensation in the epigastric region, which was relieved by taking food, but as soon as this was taken, and often even while engaged in eating, there would come an urgent desire to evacuate the bowels.

The motions were either solid or semi-solid, and containing lumps of partially digested food, and even some scarcely more than masticated. Fowler's solution was ordered for her in two drop doses a short time before meals. The medicine was continued five weeks, and a perfect recovery followed. In gastralgic and irritable neurotic conditions of the stomach I have derived much benefit from one to two drop doses of Fowler's solution taken on an empty stomach.

Acne Pustulosa.—In the clinic of Professor Duhring, Dr. A. Van Harlingen reports (Philadelphia Medical Times, Jan. 1, '72) the following case:

This disease is a chronic one. The patient tells us he has had it about a year. This fact at once excludes the idea of its being the eruption of variola, to which, at first glance, it bears a close resemblance.

Another affection for which it may be mistaken is a pustular syphilitic disease of the skin, which it also resembles. But were it the latter we should find it probably involving the scalp, which in the present instance is not the case.

Having eliminated variola and syphilis, we have but one disease to eliminate: this is acne.

We have here an aggravated form of this affection; for you will not often see a case where the disorder has made such marked progress. A peculiar feature is the age of the patient—fifty years. Acne is a disease of the young, not ordinarily occuring in persons over twenty-five. In the present instance the affection is probably due to ill health, an acne cachecticorum so-called, and if we build up this man's system we shall do much toward the cure of the disease.

The eruption is the product of a low, vitiated state of the secretions; and in this connection you will notice the tendency to the formation of small abscesses at various points, a symptom not characteristic of most kinds of acne. The one before us is the slow pustular variety, not resembling the ordinary acne punctata.

I shall order this man to apply the unguentum diachyli, of which I give you the formula, taken from Hebra, as it is not officinal, and you will not be likely to find many apothecaries who keep it on hand.

| | |
|---|---|
| R.—Olei Olivarum opt., . . . . . | oz. xv. |
| Lithargyri, . . . . . | oz. iij–dr. vj. |
| Coque, dein. addi | |
| Olei Lavandulæ, . . . . . . | dr. ij. |

Misce. Fiat unguent.

An attempt to apply any more stimulating ointment in a case of this kind would bring on new pustules, and make matters worse.

In addition to the external application, I shall order this man the following:

| | |
|---|---|
| R.—Ferri et Ammoniæ citrat., . . . | dr. j. |
| Liquor Potassæ arsenit., . . . | fdr. ss. |
| Tinct. Cinchonæ comp., | |
| Syrupi simplicis, aa. . . . . | foz. ii. |

Misce. Sig.—Teaspoonful after each meal.

The arsenic is given, not with a view to any specific effect to be expected from it, but merely as a general tonic, and, as you see, in small doses. Besides the iron and arsenic, I shall order the patient cod-liver oil.

Hypophosphites of Iron, Quinia, and Strychnia, in Cases of General Debility and Nervous Exhaustion.—J. H. H. states (Amer. Jour. Med. Sciences) the particular cases for which Dr. W. D. Day, of London, has found the hyperphosphites especially applicable, are—"I. Those of simple and complicated debility, where all the functions are working naturally, but tardily, depression of strength and spirits is a prominent manifestation. 2. Those of anæmia, with local derangement of one or more sets of nerves, as in neuralgia, or threatening palsy from nervous exhaustion, or slow blood change from the diathesis of struma, sypilitic tubercle. 3. Those especially marked by nervous exhaustion and muscular weakness, from whatever cause arising. 4. Those of excitability and irritability of the nervous system, hysteria, insomnia, some forms of epilepsy, and exhaustion of the brain from overwork, or from any of the usual causes of debility. 5. Those of atonic dyspepsia, and gastralgia from blood impoverisement, in which chalybeate preparations are indicated but, through feeble digestive powers, cannot be tolerated."—*Med. Cosmos*, *Nov.*, '71.

Celery as a Nervine.—A correspondent of the Practical Farmer says (Med. Bulletin, Cincinnati Med. Repertory) "I have known as many men and women too, who, from various causes, had become so much affected with nervousness that when they stretched out their hands they shook like aspen leaves on windy days; and by a daily moderate use of the blanched foot stalks of the celery leaves as a salad, they became as strong and steady in limbs as other people. I have known others so very nervous that the least annoyance put them in a state of agitation, and they were in almost constant perplexity and fear, who were effectually cured by a daily moderate use of blanched celery as a salad at meal times. I have known others cured by using celery for palpitation of the heart."—*Med. Cosmos*, *Nov.*, '71.

Seton Treatment of Serous Tumors.—Dr. Galloupe reports that he had operated by seton recently on about a dozen of these cases, including five or six of of hydrocele, several of weeping sinew, one hydrocele of neck, all successfully, and with little trouble.

Beef Tea.—Many persons believe that beef tea is very nourishing, and that it is an excellent strengthener for people of weak health. This is a mistake. Some few practitioners and chemists have long been aware of the fact, and now their view is confirmed by D. Marcet. There is no nourishment in beef tea. Mixed with solid food, it imparts a relish which promotes digestion; and the best solid that can be mixed therewith is the beef from which it is made, reduced to a powder. In two, at least, of the London hospitals the mixing of powdered beef with the beef tea has long been practiced, and there the patients get strong on a beef tea diet. It is worth remembering, too, that the objections to the use of beef tea apply equally to the preparation described as extract of meat, with the further disadvantage that the extract is always stale.—*Chambers' Journal and Druggists' Circular.*

Ice in Acute Rheumatism.—Professor Esmarch, in a communication to the Berlin Medical Society, related instances of the great benefit which he had derived from the continuous application of ice to joints affected with acute rheumatism. The general temperature becomes lowered, the pain abated, and the course of the disease abbreviated to an extent procurable by no other means. So far from fearing the induction of cerebral affection by repelling the articular inflammation—the *phrenopathia rheumatica* being here, as in typhus, dependent upon the increased temperature—ice is especially indicated for its prevention or removal.—*Medical Times and Gazette.*

Canula for Tracheotomy.—Dr. Benjamin Howard, of New York, (Med. Record) gives the following method for extemporizing a canula:

Take a piece of lead, whether in the form of sheet, pipe, or bullet, and, if necessary, hammer it out as thin as it can be used without breaking. Of this cut a piece the shape of a parallelogram about $2\frac{1}{2}$x$1\frac{1}{4}$ inches, or enough larger to allow a margin; roll it around a trimmed stick—ramrod or pencil—thus making a tube, and bevel both edges so that by trimming and dressing the seam may be smooth and firm. Cut the upper end so as to form four slips of equal size, and at the middle of the tube cut out a transverse elliptical section from about two-thirds of its circumference. Withdraw the pencil, and bend the tube upon itself. Turn down the slips, and in two of them cut eyelet holes, through which a tape string may be passed around the neck, to retain the canula in its position in the wound.

Lead, the material used, is particularly innocuous; and either in sheet, pipe, or bullet form, is found almost everywhere within and beyond the bounds of civilization. The tools needed are simply a smooth stone and a penknife; with these alone a canula of sufficiently finished construction may be readily prepared.

Tinctures Podophillum, Dandelion, and Phytolacca.—Dr. E. W. Knepper, in speaking of "Office Pharmacy," (Indiana Journal of Medicine) says:

Take, for instance, the podophillum; prepare a tincture from the recent root, dr. viij to the pint of alcohol, and you have a remedy that after you have once used, you will never want to be without. Dose, as an alterative, ten to fifteen drops; as a cathartic, half drachm. I am satisfied that a trial of this tincture will satisfy any one of its superiority over podaphillum. It makes no difference whether used as a stimulant to the digestive organs, as an alterative, or as a general cathartic. Or, if you wish to use the dandelion, prepare a tincture in the same way, and you have a better medicine than the taraxicum in the market. Or take the *Phytolacca decandria*, or poke root. This remedy has not, I am satisfied, received the attention and credit it deserves. It exerts a direct influence upon the processes of waste and nutrition, and therefore possesses those properties called alterative in a high degree. I have used it in scrofula, secondary syphilis, and chronic skin diseases, with excellent results. A tincture may be made in the way before mentioned.

To Administer Pills.—Some persons can swallow pills without any trouble, while with others it is almost impossible. I have tried various ways to administer them to such persons, and the following is the best plan I ever hit upon: Put the pills under the tongue and behind the teeth and let the patient immediately take a large swallow of water, and he will neither feel the pill nor taste it. In fact, he can't tell where it has gone, and I have seen them look about the floor to see if they hadn't dropped it.—*Chicago Med. Times.*

Treatment of Ozæna.—Dr. T. C. Henry (Cincinnati Lancet and Observer) says: "In the treatment of ozæna, the bichloride of mercury will be found useful; also carbonic acid as a disinfectant. In number three of the Journal of Opthalmology and Otology, several instances are reported, in which no trifling harm was effected by the employment of Thudicum's nasal douche. The posterior nasal syringe is by far the safest instrument in treating diseases of the nasal cavities, and sufficiently effectual.

Alcohol.—The following "declaration" respecting alcohol has recently been published in the British medical journals. It is signed by two hundred and fifty-four physicians and surgeons, including some of the most distinguished names in the profession in Great Britain. Its appearance naturally excites much criticism.—*Boston Med. and Surg. Journal.*

As it is believed that the inconsiderate prescription of large quantities of alcoholic liquids by medical men for their patients has given rise, in many instances, to the formation of intemperate habits, the undersigned, while unable to abandon the use of alcohol in certain cases of disease, are yet of opinion that no medical practitioner should prescribe it without a sense of grave responsibility. They belive that alcohol, in whatever form, should be prescribed with as much care as any powerful drug, and that the directions for its use should be so framed as not to be interpreted as a sanction for excess, or necessarily for the continuance of its use when the occasion is past.

They are also of opinion that many people immensely exaggerate the value of alcohol as an article of diet, and as no class of men see so much of its ill effects, and possess such power to restrain its abuse, as the members of their own profession, they hold that every medical practitioner is bound to exert his utmost influence to inculcate habits of general moderation in the use of alcoholic liquids.

Being also firmly convinced that the great amount of drinking of alcoholic liquors among the working classes of this country is one of the greatest evils of the day, destroying—more than anything else—the health, happiness and welfare of those classes, and neutralizing, to a large extent, the great industrial prosperity which God has placed within the reach of this nation, the undersigned would gladly support any wise legislation which would tend to restrict within proper limits the use of alcoholic beverages, and gradually introduce habits of temperance.

Charcoal in Gastric and Enteric Disorders.—Dr. Remy highly recommends the use of vegetable charcoal prepared from the poplar after the process of M. Belloc. He exalts its efficacy as "truly marvellous" in the treatment of gastralgia, gastro-enteralgia, dyspepsia, pyrosis, the greater number of nervous affections of the stomach and bowels, and constipation. In certain cases of dysentery it has also been found very useful, and in one such instance, reported by Dr. Farr, of London, its effect was said to be very satisfactory in the form of enemata.—*L'Union Medicale.*

Splenitis and Chronic Ague.—In a case of this character Professor N. S. Davis (Chicago Medical Examiner, Feb. 1, '72) remarks:

The patient was directed to have five grains of sulphate of quinine, with two of blue mass, each morning and noon for two days, then the quinine alone every morning for a week or more, at the same time he was to take ten grains of hydrochlorate of ammonia, dissolved in liquorice, every four hours until the splenitic enlargement disappeared—the latter was said to be an old remedy for visceral enlargements consequent on chronic ague, it having been recommended by Dr. Eberle in his work on practice. After the intermittent paroxisms have ceased, and the local symptoms of inflammation have abated or disappeared, it will be proper to keep the patient for some time on small doses of quinine in combination with a soluble salt of iron, of which the citrate or the phosphate are the best. This combination should be continued, with a mild, easily digested diet, and moderate out of door exercise, until the patient regains his muscular vigor, and the blood its natural proportion of red corpuscles.

Professor Sayre's Vertebrated Probe and Catheter. In the British Medical Journal for July 22, '71, Professor Sayre, of New York, describes the vertebrated probe and catheter which he has devised, as follows:

It consists simply of a series of hollow silver disks, made a trifle smaller at one end than at the other, so as to fit into one another, like a pile of cups or tumblers. These are held together by a linked chain running through the series, and jointed nearly opposite each disk insertion. The chain terminates in a square rod which runs through the last disk, and is much larger than any of the other; and on the end of the small rod is cut a thread, on which runs a small button screw, which can make the chain tight or loose at pleasure. Of couse when the screw is turned back, the chain being lengthened, the disks fall away from one another, and the probe is as limber as a chain, capable of following any sinuosity into which it may be pushed; and by a few turns of the screw, the chain being shortened, the disks are drawn firmly together, so as to make a solid probe, which will give the concussion against carious or necrosed bone, the same as any other probe. A small slot is made in the canula containing the screw, for the purpose of putting a small nut which regulates the tension of the chain, and thus prevents the possibility of applying any sufficient force to break it. There are two fenestræ at the distal disk, for the purpose of drawing an oakum seton through deep sinuses and carious joints; this makes it also very useful as a catheter in cases of tortuous urethra from enlarged prostate. It is impossible to make a false passage with it, and, as it is simply a series of ball and socket or universal joints, it will follow any passage, however devious. By simply unscrewing the steel bulb at the end, and inserting a bulb of porcelain, according to the suggestion of Nelaton, you have the most perfect bullet probe that can be desired.

To clean it, it is necessary to unscrew it at the end, and to remove the small screw in the slot in the canula, when it will immediately fall to pieces. After washing, it is easily put together, just the same as a string of beads, only remembering to put the small end at the disk on the wire first; and, as each disk increases in length until the end, of course no error can occur in making them fit properly.

The description is illustrated by two wood cuts.—*Med. Times*, *Oct.* 16, '71.

Sulpho-Carbolate of Soda.—Dr. J. B. Crawford (Proceedings Luzerne County Medical Society—Cincinnati Medical News, Feb. '72) says:

Among the antiseptics which are capable of being absorbed into the blood, carbolic acid holds a prominent place. Its disagreeable taste and odor and its caustic action, when uncombined, render it very objectionable in its pure state. The sulpho-carbolate of soda furnishes a form for administering it which is free from these objections. This salt contains one-fourth of its weight in carbolic acid. As high as sixty grains have been given to an adult, repeated at intervals of four hours, being the equivalent of fifteen grains of carbolic acid, or ninety grains in twenty-four hours; a quantity far exceeding in amount that which could be given in a crude form.

The fact that in cases where a larger amount of this salt has been administered, sodium sulphate has been found in the urine, but no trace of carbolic acid, while the breath of the patient presented a strong odor of carbolic acid, would seem to indicate that the salt was decomposed in the tissues of the blood, and that carbolic acid, thus set free, must be brought into direct centact with any living organisms contained in the tissues of the blood. This would seem to satisfactorily explain its asserted virtues in the treatment of zymotic diseases. My own experience in the use of this article has not been extensive, yet the results obtained have been very satisfactory. I have treated about thirty cases of scarlatina during the past year, in which I have relied almost exclusively upon this article. Most of my cases were mild, although several of them were severe. All recovered. I have administered it in about fifteen cases of diptheria, and, whenever given early, with equally satisfactory results. I applied a strong solution (dr. i ad oz. ij) to a troublesome case of barbar's itch, and soon effected a cure. Within a few days I have seen a chancre rapidly heal, to which only lint, saturated with a solution of this substance, was applied.

INDICATIONS FOR THE EMPLOYMENT OF THE CATHETER IN OLD PERSONS.—M. Guyon, in one of his clinical conferences at the Hopital Necker, lately remarked that retention of the urine is very common in old men, depending generally on affections of the bladder, or of the neck of the bladder, or of the prostate. Many cases of supposed vesical paralysis are in reality due to prostatic disease. Retention of urine in old people displays itself by symptoms that are eminently variable. Some of these symptoms are strongly marked: the patients require to micturate frequently, and in doing so experience pain and burning heat which lasts for a long time; there may even be constitutional and febrile symptoms. In other instances, again, the symptoms are by no means prominent, especially in those cases where the bladder is but little contractile; the retention is only then indicated by percussion, palpation, and catheterism, the latter alone, in many cases, being reliable evidence of its presence. But this indication that catheterism should be adopted as an exploratory means is somewhat delicate, for the operation is not always inoffensive, and the patient suffering but little, subsequent troubles may be attributed by the patient or his friends to the injudicious interference of the surgeon. If, however, the symptoms be well marked, then there is no room for hesitation, and M. Guyon even goes so far as to say that the catheter should be passed in the case of every old man who evacuates the contents of his bladder imperfectly. He thinks that it is not necessary that it should enter the organ on the first occasion, since, if only introduced as far as the neck, it habituates the tissues to the contact of instruments, and indicates, in part at least, the seat of the disease. Stoppage of the flow cf water is always a serious symptom in old people, and the best advice that can be given to them is to be sounded, either with a simple sound or a catheter, and that frequently. Indeed, if relief be not speedily afforded to such patients, dangerous symptoms soon make their appearance in the form of rigors, purulent urine, and violent reaction.

Purely medical treatment is of no service in such cases.

Not only stoppage of the flow of water occasions grave accidents, but it stimulates other diseases; it causes alterations of the walls of the bladder, and provokes cystitis. When the bladder is greatly distended, however, it is imprudent to evacuate it completely. The frequency with which catheterism should be repeated is an important question. No absolute rule can be laid down, but it may be performed every five hours; but commonly the instrument should only be passed when there is an intense desire to urinate. If, however, he experience but little or no inconvenience, it should be passed at regular intervals. As a rule, the permanent retention of the catheter in the bladder is to be avoided, except perhaps in cases when the the desire to pass water is very intense and frequent, or when the introduction of the catheter is very difficult. M. Guyon cites a case where it was worn for two years. It should in general be fixed in position till the bladder is habituated to catheterism. As adjuvants to the above treatment, injections may be employed, which may be hot, cold, or medicamented, as occasion may require.—*London Practitioner, Jan.* '72.

Vomiting of Pregnancy Treated by Electricity.—Dr. S. Iffla presented a memoire to the Medical Association of Victoria, Australia, communicating the great value of electricity in cases of obstinate vomiting of pregnancy. In one remarkable case, defiant of all medication, where the patient was reduced to the last degree, prompt recovery followed the application of the constant current to the epigastric region. A recurrence of the same difficulty in the same case in a subsequent pregnancy as promptly yielded a second time to the same means.—*Bull. Therap. Dec.* 15, '71.

Chloral in Gout.—Chloral must be an efficient remedy for this disease, as Dr. Pugliese (Lyon Medicale, No. 21) says that he gave thirty grains of it to a patient suffering horrible agony, "never closing an eye from September 25th to October 3d, and ten minutes afterward he fell asleep to awake in a condition of "beatitude indiscriptible."

Chloride of Ammonium in Hepatitis and Hepatitic Abscess.—This remedy is highly lauded in all cases of hepatic derangement where mercury or other resolvents are indicated, both in France and Germany. It is given in doses of one gramme (15 gr.) morning and evening.—*Bull. Therap., Dec.* 15, '71.

